# Editorial: Genetic/epigenetic mechanisms and related clinical strategy in leukemia

**DOI:** 10.3389/fonc.2023.1275992

**Published:** 2023-08-21

**Authors:** Yonghui Li, Liping Dou, Clara Nervi

**Affiliations:** ^1^ Central Laboratory, Shenzhen Key Laboratory of Precision Medicine for Hematological Malignancies, Shenzhen University General Hospital, Shenzhen University Health Science Center, Shenzhen, Guangdong, China; ^2^ Department of Hematology, People’s Liberation Army General Hospital, Beijing, China; ^3^ Department of Medical and Surgical Sciences and Biotechnologies, Sapienza University of Rome, Rome, Italy

**Keywords:** leukemia, epigenetics, genetics, t(8;21) AML, miRNAs, COVID-19

Leukemia is a kind of hematologic malignancy feathered by unlimited rapid proliferation and disordered differentiation of hematopoietic blast/progenitor cells (1). In the past decades, unprecedented progress has occurred in the biology of leukemogenesis and clinical management of leukemia with new tools for detection and multiple treatment options appearing. However, patients diagnosed with leukemia nowadays still have poor outcomes and high mortality rates. The newest cancer statistic report demonstrated that the number of estimated new cases and deaths of leukemia ranked among the top ten of all cancers (2). Primary chemoresistance and relapse become difficult problems in the treatment of leukemia. Consequently, the detection of robust biomarkers and individual precision medicine are in urgent need for leukemia. In the Research Topic *Genetic/epigenetic mechanisms and related clinical strategy in leukemia*, we received 4 articles on the prognosis, prediction and treatment of leukemia.

Studies from Yu et al. and Zhou et al. were about the prediction of prognosis in acute myeloid leukemia (AML). Yu et al. used the public datasets to explore differentially expressed genes between AML patients and healthy cohorts and established a novel risk scoring system, which was proved to be effective in the prediction of AML prognosis in multiple datasets. It might be used as a supplement to the commonly used National Comprehensive Care Network (NCCN) risk stratification tool, as it could furtherly divide the intermediate group into relatively low-risk and high-risk groups. Karyotype is an essential factor for the prognosis of AML. Zhou et al. did a multicenter retrospective study and explored the risk factors for post-transplant relapse and survival in younger adult patients with t(8;21)(q22;q22) AML undergoing allogeneic hematopoietic stem cell transplantation (HSCT). Results showed that patients undergoing transplantation in CR1 had favorable survival. Measurable residual disease (MRD) monitoring in the first 3 months after allo-HSCT might be robust in predicting relapse and adverse survival after allo-HSCT. It highlighted the importance of MRD quantification in the prognosis of AML. Different techniques for MRD assessment are also an area worthy of much study (3).

The study by Wu et al. depicted the profile of miRNAs and their interfering targets in peripheral blood mononuclear cells from patients with chronic myeloid leukemia (CML). They reported the differentially expressed miRNAs (DE-miRNAs) were significantly related to vital biology functions such as cell cycle, cell differentiation, and apoptosis: DE-miRNAs could predict the prognosis of CML patients. Regarding the treatment of CML, Yu et al. conducted a comprehensive review about the management of CML patients with COVID-19 and hepatitis B because TKI treatment might impair the immune function.

The exploration of the genetic/epigenetic mechanism of leukemogenesis forms the basis of new treatment development. For example, the discovery of pathogenic gene mutation promoted the progress of targeted therapy such as FLT3 inhibitors and IDH1 inhibitors (4). An aberrant epigenetic profile was found to contribute to the occurrence of leukemia and hypomethylating agents were subsequently proved useful in the treatment of leukemia (5). On the one hand, previously poor risk stratification might transform into a relatively better prognosis due to new targeted therapies. On the other hand, distinguishing patients who are more suitable for a targeted therapy facilitates the establishment of a more detailed risk stratification system. Traditional chemotherapy, immunotherapy, targeted therapy, and transplantation can be properly combined to treat AML, and it is crucial to choose the best therapy for an individual patient.

The original articles and reviews collected in this Research Topic discussed the risk stratification and prognosis prediction of leukemia from various perspectives. The prognostic factors include both clinical characteristics and molecular changes, both genes and miRNAs. As for the methodology, retrospective clinical analysis and bioinformatics were both used. However, more research is needed, especially on the genetic/epigenetic mechanism of leukemogenesis, which may lead to the finding of novel prognostic biomarkers and therapeutic targets to improve AML outcomes.

## Author contributions

YL: Conceptualization, Funding acquisition, Investigation, Writing – original draft, Writing – review & editing. LD: Conceptualization, Validation, Writing – review & editing. CN: Conceptualization, Funding acquisition, Supervision, Writing – review & editing.
